# Optic nerve sonographic parameters in idiopathic intracranial hypertension, case-control study

**DOI:** 10.1038/s41598-024-85033-4

**Published:** 2025-01-13

**Authors:** Islam El Malky, Wael Elshazly Aita, Alaa Elkordy, Marwa Nasreldeen, Mahmoud Abdelhafiz, Amr M. Tael, Hazem Mo. Abdelkhalek

**Affiliations:** 1 Department of Neurology, Director of Interventional Neurovascular Unit, South Valley University, Qena, Egypt; 2 MD Ophthalmology, Department of Ophthalmology, South Valley University, Qena, Egypt; 3 MD Neurology, Department of Neurology, Tanta University, Tanta, Egypt; 4 MD Anesthesiology, Department of Anesthesiology, South Valley University, Qena, Egypt; 5 MD Neurology, Department of Neurology, South Valley University, Qena, Egypt; 6 MD Neurosurgery, Department of Neurosurgery, South Valley University, Qena, Egypt; 7 Assistant Professor of Neurology, Department of Neurology, Tanta University, Tanta, Egypt

**Keywords:** IIH, ICP, Headache, ONSD, Transorbital ultrasonography, Neuroscience, Diseases, Medical research, Neurology

## Abstract

The most common diagnostic error of IIH is inaccurate funduscopic examination. Moreover, IIH could be diagnosed without papilledema. Trans orbital sonography could be used as a non-invasive and cheap tool for discovering increased ICP (intracranial Pressure). Aim of our study was discovering the changes in ultra-sonographic indices and which one could predict the increased ICP. Sixty-eight patients were diagnosed as definite IIH and 68 healthy volunteers are included in the study who had the same sex and age. ONSD, peak systolic velocity (PSV), end diastolic velocity (EDV), and resistance index (RI) were estimated by transorbital color Doppler. Multivariate linear regression was used to discover the predictors of increased ICP. ROC curve was plotted for the predictor. A statistically significant difference was found between IIH patients and controls regarding ONSD, EDV and RI. Multivariate linear regression revealed that ONSD is the only predictor of increased CSP pressure. Its cut-off value indicating high ICP was 5.7 mm on Rt and Lt eye (AUC: 0.916; 95% confidence interval 0.867–0.965; *p* < 0.001; 90% sensitivity, 80% specificity at Rt eye. AUC: 0.902; 95% confidence interval 0.845–0.958; *p* < 0.001; 91% sensitivity, 80% specificity at Lt eye).

## Introduction

Idiopathic intracranial hypertension (IIH) is diagnosed by generalized headache with blurred vision due to papilledema in the absence of an identifiable cause, such as cerebral venous sinus thrombosis or brain masses in MRI. The diagnosis is established by an increased opening pressure of cerebrospinal fluid (CSF) (> 25 cm H2O)^1^. The prevalence is 0.9 cases per 100,000, but more common 20 times in obese women during the childbearing period^2^. The underlying cause of IIH is still largely unknown. However, IIH is frequently misdiagnosed, and nearly 39.5% of patients referred to headache clinic^3^. The most common diagnostic error is inaccurate funduscopic examination^3^, which can be challenging^4^. Moreover, IIH could be diagnosed without papilledema^5^.

Optic nerve sheath diameter is the subarachnoid space around the optic nerve, which becomes full of CSF and dilated in IIH, leading to papilledema and visual affection^6^. Measuring ONSD by ultrasonography was a non-invasive and inexpensive tool for discovering increased ICP (Intracranial Pressure) in comparison to expensive one like magnetic resonance imaging (MRI)^7^. Hemodynamic changes of the central retinal artery are expected in cases with IIH, which have been studied in a few publications^8,9^. Our study aims to discover the cut-off values of ONSD and the changes in PSV (Peak systolic velocity), EDV (End Diastolic velocity), and RI (Resistance Index) in IIH patients and to discover parameter that predict increased CSF opening pressure.

## Methodology

Sixty-eight patients were diagnosed with a definite IIH (CSF opening pressure of more than 250 mmH2O), according to the International Classification of Headache Disorders, 3rd edition^1^. Sixty-eight participants without attacks of headache or signs of increased ICP at the time of the study were included as a control group with the same sex and age, identical to the patients’ group. The study was approved by the local ethics committee of our university (South Valley University, Egypt) with a number (SVU/MED/NAP020/4/21/12/280) and performed according to the Declaration of Helsinki. Written informed consent was obtained from all subjects before enrollment in the study. The study was held in our headache clinic from December 2021 to December 2023.

Inclusion criteria for participants (patients and controls) were ≥ 18 years old without neurological or medical conditions such as hypertension, renal, or hepatic diseases. All participants have normal MRI and MRV brain except for signs of increased ICP such as empty sella turcica, dilated optic nerve sheath, flattening posterior aspect of the globe, and transverse venous sinus stenosis in IIH group. Exclusion criteria included patients with underlying causes, such as venous-sinus thrombosis or intracranial masses or patients with a contraindication of LP puncture, such as intracranial mass, blood coagulopathy, and skin infection. Lumbar puncture was performed with the subject (patient or control) as the gold standard for CSF pressure measurement in the lateral decubitus position without sedative medications.

Demographic data of all participants, such as age, sex, presentations, marital status, history of recurrence, family history, and BMI (Body Mass Index), was collected. A neuro-ophthalmological history and examination were reported in detail for all patients and controls. All symptoms and/or signs were recorded, such as headache, blurred vision, papilledema, and 6th nerve palsy.

Two investigators trained in ocular sonography carried out an ultrasound B-mode and color mode eye examination using previously established techniques^10^. A Samsung HS40 machine was used with Linear probe (LA3-16 A). All patients and controls were examined in a supine position with a head elevation of 20–30º. The mechanical index (MI) was reduced to 0.2 to avoid bio-thermal side effects of the ultrasonography. A thick layer of ultrasound gel will be put on the closed eye, protected by a transparent cover. The ultrasound probe will be placed axially on the upper eyelid of the eye globe. The papilla and the optic nerve, along its longitudinal course, will be visible in a transversal plane where the anterior portion of the optic nerve is located. ONSD was measured three millimeters beyond the retina on an axis perpendicular to the optic nerve. The distance between the hyperechogenic dura mater and periorbital fat, which encircles the hypoechogenic subarachnoid space, was used to measure ONSD. This space is well delineated by hyperechogenic dura mater and periorbital fat.

**Fig. 1:**
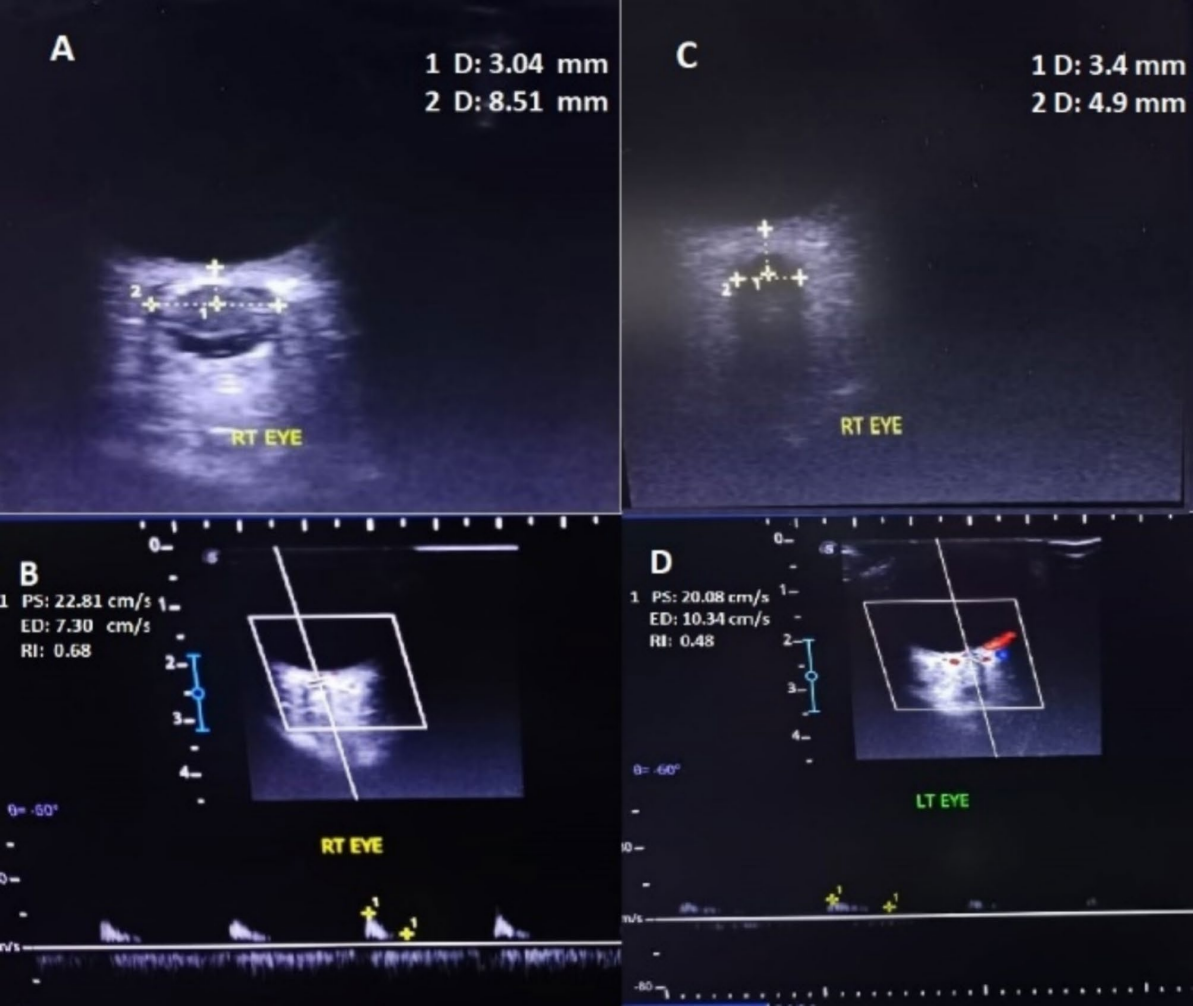
Trans-orbital Sonography in a patient with IH and control. (**A**, **B**) of IIH patient with papilledema reported increased ONSD = 8.5 mm with decreased EDV (7.3 cm/s) and increased RI (0.7) in comparison to normal control (**C**, **D**) who reported ONSD = 4.9 mm with EDV (10.3 cm/s) and RI (0.5).

All color Doppler parameters, such as Peak systolic velocity (PSV), end-diastolic velocity (EDV), and Resistance index (RI), were recorded manually. This was repeated three times to ensure the accuracy of the recording. The central retinal artery was targeted three mm from the globe **(**Fig. 1**)**.

## Statistical analysis

Sample size was calculated according to the following formula: n = (Z_α/2_+Z_β_)^2^ *2*σ^2^ / d^2^ (1.94 + 0.84)^2^ *2*1.34 ^2^ m.m / 1.2 ^2^ m.m ) so, the minimal sample size for participants was 50 in each group. We used SPSS (version 28), and a p-value less than 0.05 was considered significant. Age and PSV (Rt and Lt) were reported as mean ± SD, while BMI, ONSD, RI and EDV were reported as median and range. Statistical analysis was performed with the student’s independent t-test or Mann-Whitney according to variable type. Multivariate linear regression was performed to discover the predictors of increased ICP. Receiver operating characteristic (ROC) curves were plotted for ONSD, and the area under the curve (AUC) was estimated to assess the diagnostic accuracy for increased ICP.

## Results

### Demographic data

Sixty-eight IIH patients were recruited prospectively in our study, also the same number of healthy volunteers as a control group. The IIH group had 65 female patients (97%), with a mean age of 30.22 ± 9.93, while the control group had 63 female participants (94%) with a mean age of 31.96 ± 9.43. Fifty patients (74%) were married in the IIH group, while 58 (86.6%) were in the control group. Forty-six patients (68.7%) had kids in the IIH group, while 53 participants (53.3%) were in the control group. There was no statistical difference in sex, age, marital state, or having kids between both groups (p-value = 0.405, 0.446, 0.082, and 0.170, respectively). The control group had infrequent migraine attacks in five patients (7.4%) but without any symptoms or signs of increased intracranial pressure at the time of the study.

The BMI median of the IIH group was 30.819 (Max-Min BMI: 57.024–20.761), while in the control group was 27.531 (Max-Min BMI: 47.653–18.929), with a statistically significant difference (p value = 0.003). IIH patients had headache and papilledema in all patients, tinnitus in four patients (6%), blurred vision in 52 patients (77.6%), and six nerve palsy in eight patients (11.9%). Recurrent IIH was reported in nine patients (13.4%), and positive family history was noticed in six patients (9%). CSF pressure mean in IIH group was 35.61 ± 4.885, while it was 19.81 ± 2.607 in the control group, with a statistically significant difference (p-value = 0.001).

### Transorbital sonography

The sonographic data of our study is reported in (Table 1**)**. Both groups had a statistically significant difference regarding ONSD, EDV, and RI at both eyes but no statistically significant difference regarding PSV.

**Table 1:** The sonographic data.

Variable	IIH group	Control group	*p*-value
**Rt eye**			
ONSD (mm)	Median 6.52	Median: 5.2	0.0003*
	Range (8.5–4.47)	Range: (6.46–3.67)	
PSV(cm/s)	Mean ± SD: (20.75 ± 7.89)	Mean ± SD: (22.24 ± 4.39)	0.169
EDV (cm/s)	Median 7.3	Median 9.1	0.001*
	Range (12.78–2.04)	Range (16.32–5.48)	
RI	Median 0.63	Median 0.57	0.001*
	Range (0.89–0.39)	Range (0.42–0.74)	
**Lt eye**			
ONSD (mm)	Median: 6.7	Median: 5.28	0.001*
	Range: (8.3–4.36)	Range: (7.14–3.61)	
PSV (cm/s)	Mean ± SD: (21.01 ± 6.44)	Mean ± SD: (21.13 ± 3.64)	0.846
EDV (cm/s)	Median 7.69	Median 9.1	< 0.001*
	Range (11.86–1.5)	Range (12.78–5.48)	
RI	Median 0.65	Median 0.57	< 0.001*
	Range (0.85–0.41)	Range (0.74–0.33)	

ONSD (Optic Nerve Sheath Diameter), PSV (Peak systolic velocity), EDV (End Diastolic velocity) and RI (Resistance Index). (*): Statistically significant.

A multivariate linear regression test between these variables and CSF pressure measures was performed to detect the most predictive variable. The test was performed separately on Rt and Lt eye measures to avoid multicollinearity. The test revealed that ONSD was the only predictive variable of CSF pressure measurement bilaterally (Rt and Lt eyes) (Table 2).

**Table 2:** Multivariate linear regression test.

	Standardized coefficients	*P*-value.	95.0% Confidence Interval for B	
			Lower bound	Upper bound
RT Eye				
ONSD	0.668	0.000*	4.942	7.724
EDV	−0.01	0.929	−0.63	0.576
RI	0.053	0.528	−10.3	19.93
Lt Eye				
ONSD	0.603	0.00*	3.972	6.611
EVD	−0.02	0.863	−0.67	0.797
RI	0.148	0.128	−3.57	28.08

ONSD (Optic Nerve Sheath Diameter), PSV (Peak systolic velocity), EDV (End Diastolic velocity), RI (Resistance Index), RT (Right) and Lt(Left). (*): Statistically significant.

**Fig. 2:**
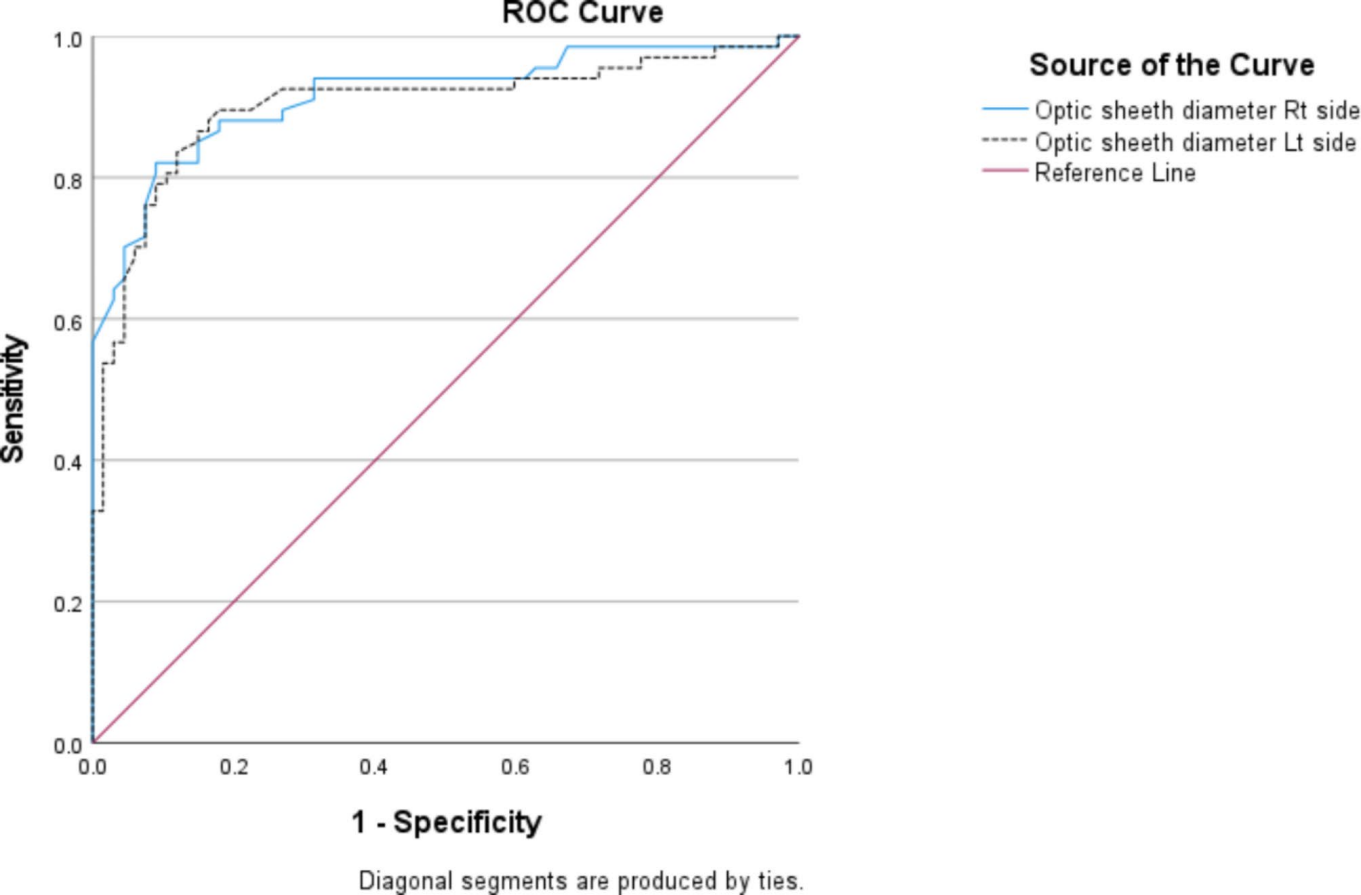
ROC curve of optic nerve sheath diameter for both eyes related to increased intracranial pressure. The ONSD cut-off value were 5.68 mm and 5.69 mm on Rt and Lt eye respectively (AUC: 0.916; 95% confidence interval 0.867–0.965; *p* < 0.001; 90% sensitivity, 80% specificity at Rt eye. AUC: 0.902; 95% confidence interval 0.845–0.958; *p* < 0.001; 91% sensitivity, 80% specificity at Lt eye).

ROC curve analysis revealed the best cut off value of ONSD for prediction of increased ICP at 5.7 mm on both eyes (AUC: 0.916; 95% confidence interval 0.867–0.965; *p* < 0.001; 90% sensitivity, 80% specificity at Rt eye. AUC: 0.902; 95% confidence interval 0.845–0.958; *p* < 0.001; 91% sensitivity, 80% specificity at Lt eye) (Fig. 2).

## Discussion

In this prospective study, we reported the importance of ONSD in diagnosing IIH. The study revealed the cut-off value of ONSD in IIH patients at 5.7 mm. The importance of using ultrasonography to determine ONSD as a diagnostic tool for increased ICP appears in the differentiation between cases with papilledema and pseudo-papilledema, in addition to cases with difficult fundus examination such as opaque lenses or vitreous problems^11^.

Our cut-off value of ONSD is in concordance with other studies^10,12,13^, which reported ONSD between (5.6–5.9 mm), indicating reproducibility and accuracy of ONSD measuring by trans-orbital ultrasonography in the diagnosis of increased ICP. Other studies revealed different cut-off values of more than 6 mm^14,15^. These different measures result from the usage of different devices and parameters between different studies in addition to performance on different patients such as neuro-critical patients of brain trauma, ICH (intracerebral hemorrhage), or infection who are usually sedating or mechanically ventilated^16,17^. Also, we cannot neglect the differences in ethnicity between studies. Mehrpour M. et al. from Iran reported cut-off value of 5.5 mm while Wang L. et al. from China reported 4.1 mm^18,19^. Our study included Egyptian patients, who had similar results to other Egyptian studies, such as Razek AA et al. who reported a cut-off value of ONSD 5.45 mm in a similar population study IIH patients^20^.

CRA is a branch of the ophthalmic artery, piercing the optic nerve sheath 6–8 mm behind the globe. It gets out as an independent branch or in common with one of the posterior ciliary arteries. It has three parts: intraorbital (below the Optic nerve ON), intravaginal (the space between the ON and its sheath), and intraneural (inside the ON)^21^. Hemodynamic changes of the central retinal artery are expected in cases with IIH, which have been studied in a few publications^8,9^. Increased RI means an increase in the resistance of the vessel and a decrease in its compliance, which is expected with increased CSF pressure around the CRA inside the enclosed subarachnoid space, leading to decreased blood velocities. Our study reported a statistically significant decrease in EDV and an increase in RI but no statistically significant decrease in PSV. Querfurth HW et al. reported a similar decrease in EDV but without a change in RI in IIH patients^9^. In contrast to our study, other studies reported increased PSV^12,20^. The cause of that difference from our data is unclear. It is well known that a wide range of expected values in normal population are discovered in the literature. For example, the normal value of the PSV of CRA is measured to be 8.8–17.3 cm/s which is less than our healthy control group^22^. The high variability may result from differences in the device, techniques and usage of angle correction and its degree. Also, the discrepancy may be due to a low signal-to-noise ratio, as blood flow velocities are very low. Normal values are, therefore, non-replicable, and in order to enhance device-specific normal values, each laboratory must construct standardized equipment, settings, and examination procedures.

According to our knowledge, we presented in our study for the first time the regression analysis for multiple variables of increased ICP to predict the predictor. The only predictor was ONSD. Although Our study is the second largest population of adult IIH patients after Kishk NA. et al. but our study characterized by that all IIH cases were definite, included both sex, with equal number between IIH patients and control groups, and lumber puncture was performed for both groups^23^. Our study had a limitation that we didn’t follow the cases by transorbital sonography after treatment of IIH to discover the difference.

## Conclusion

Trans orbital ultrasonography Indices such as ONSD, EDV and RI might be affected in IIH patients. ONSD was the only predictor of increased ICP, with a cut-off value of 5.7 mm.

## Data Availability

The data that support the findings of this study are available from our university, but restrictions apply to the availability of these data, which were used under licence for the current study and so are not publicly available. The data are, however, available from the authors upon reasonable request and with the permission of our university.

## Abbreviations

ICP: Intracranial pressure
IIH: Idiopathic Intracranial Hypertention
ONSD: Optic Nerve Sheath Diameter
PSV: Peak Systolic Velocity
EDV: End Diastolic Velocity
RI: Resistance Index
MI: mechanical Index
BMI: Body Mass Index
ROC: Receiver operating characteristic
AUC: area under the curve
